# Exploring the mechanism and experimental verification of puerarin in the treatment of endometrial carcinoma based on network pharmacology and bioinformatics analysis

**DOI:** 10.1186/s12906-022-03623-z

**Published:** 2022-06-07

**Authors:** Zhiheng Lin, Xiaohui Sui, Wenjian Jiao, Ying Wang, Junde Zhao

**Affiliations:** 1 Shandong University of Traditional Chinese Medicine, Jinan, 250014 Shandong China; 2 Shandong University Cheeloo College of Medicine Laboratory of Basic Medical Sciences, Jinan, 250014 Shandong China

**Keywords:** Puerarin, UCEC, Network Pharmacology, Bioinformatics Analysis

## Abstract

Endometrial carcinoma is one of the two cancers with rising mortality and morbidity in recent years. In the light of many controversies about its treatment, it is urgent to construct a new prognostic model and to find out new therapeutic directions. As a small drug molecule widely used in clinical treatment and experimental research in China, puerarin has recently been proven to have obvious anti-cancer effects in multiple cancer cells. In this study, bioinformatics analysis and experimental validation were used to explore the potential mechanism of puerarin for endometrial carcinoma and construct a prognostic model. A total of 22 drug-related differential genes were found by constructing a database of drug targets and disease genes. The protein–protein interaction network was constructed for GO and KEGG enrichment analysis to initially explore the potential mechanism of its therapeutic effects. To construct the prognostic model, validation was performed by risk regression analysis and LASSO analysis. Finally, two prognostic genes—PIM1 and BIRC5 were determined to establish high and low risk groups. Kaplan–Meier analysis displayed a higher survival rate in the low-risk group than in the high-risk group. ROC curves indicated the stable and good effect in prediction (one-year AUC is 0.626; two-year AUC is 0.620; three-year AUC is 0.623). The interrelationship between immunity and its disease was explored by immune infiltration analysis. Finally, the potential effect of puerarin on endometrial carcinoma cells was further verified by experiments.

## Introduction

Endometrial carcinoma is the most common tumor in female genital tumors, occurring mostly in menopausal women aged 60 + on average [1]. According to the American Cancer Association, about 66,570 new endometrial carcinoma cases were diagnosed in the United States in 2021, with 130,000 women who died of endometrial carcinoma. Endometrial carcinoma is one of the two cancers with rising mortality and morbidity in recent years [2], which seriously affects the quality of life and the health of patients. Endometrial carcinoma occurrence is associated with excessive estrogen secretion [3]. Endometrial carcinoma is an estrogen-dependent tumor, accounting for 80% of newly diagnosed endometrial carcinoma cases. The risk of disease increases with the duration of estrogen use and remains years after estrogen withdrawal [4, 5]. At present, the endometrial carcinoma is mainly subject to surgical treatment, such as total hysterectomy, bilateral tubal ovariectomy, paraaortic and pelvic lymphadenectomy. It can also be treated through adjuvant therapies such as radiotherapy, chemotherapy and hormonal therapy [6]. There are still many controversies in the treatment of endometrial carcinoma, including the evaluation of lymph and the selection of adjuvant treatment. There is relatively little space for treatment options for advanced as well as metastatic tumors [7]. Considering the controversial and limited treatment methods of endometrial carcinoma, it is particularly important to explore the small molecule drugs for treating endometrial cancer, and to establish a new development model to accurately and conveniently predict the survival cycle of endometrial carcinoma patients.

Studies show that traditional Chinese medicine has an overall macro-control effect on disease, and has an obvious curative effect on disease control and prevention [8]. With their potential tumor selectivity and low cytotoxicity, many natural small-molecule substances present in TCM have attracted wide attention in the research and development of tumor drugs [9]. As a bioactive small molecule of isoflavone glycoside extracted from pueraria lobata, puerarin has bio-functional activities such as promoting bone formation, protecting cardiomyocytes, protecting nerve function, anti-inflammatory and alleviating pain [10–15]. Puerarin has been widely used in experimental research and clinical treatment. Puerarin injections made from puerin have been widely used in China [16]. More studies have shown that puerarin also shows significant anticancer effects in multiple cancer cell lines, such as prostate, bladder, colon, breast, and cervical cancer [17–21]. However, puerarin has not been intensively studied in endometrial carcinoma treatment. In this context, network pharmacology and Bioinformatics Analysis were used to to explore the intrinsic mechanism of puerarin in the treatment of endometrial carcinoma, establish the prognostic model of drug-related differential gene, and validate it with cellular experiments.

## Materials and methods

### Obtain the dataset of drug target genes

First, 55 drug targets related to puerarin pharmaceutical molecules were searched in TCMSP database (Traditional Chinese Medicine Systems Pharmacology). The uniport database was used to transform and standardize Puerarin drug target data to construct a target drug gene dataset.

### Construct the dataset of disease genes

Gene expression quantification RNA-Seq (SeqFPKM) and clinical data of Uterine Corpus Endometrial Carcinoma (UCEC) were downloaded from The Cancer Genome Atlas (TCGA) website (https://portal.gdc.cancer.gov). Twenty-three normal group samples and 552 tumor group samples were obtained. Data were extracted and standardized using R language (R 4.1.1). Differential genes between normal and tumor samples were screened using the ‘limma’ R package. Screen parameter setting: P < 0.05,PDR < 0.05, ∣LOGFC∣ > 1. Differentially expressed genes (DEGs) for endometrial carcinoma were obtained.

### Obtain the drug related DEGs (DR-DEGs) and construct a composite network of drug targets

Venny2.0 was used to take the intersection between differentially expressed genes and drug target genes. A total of 22 DR-DEGs intersection genes related to the differential expression of drug targets and endometrial carcinoma were obtained. Drug disease composite target network was constructed by Cytoscape (version 3.7.2), and the gene relationship network map was constructed using the “igraph”, “reshape2” R package.

### Construct and analyze a protein–protein interaction network

String database (https://string-db.org/) was used to construct a drug-differential gene protein–protein interaction network. “clusterProfiler” and “org.Hs.eg.db” in R package were used to perform GO analysis on drug related genes-DEGs, including biological processes, cellular components, and molecular functions. The same tools were used to perform KEGG enrichment analysis on DR-DEGs.

### Establish and verify DR-DEGs prognostic model

perl-based language (perl 5.28.1) was used to combine DR-DEGs expression with the clinical data to remove samples with incomplete clinical data and a survival time of 0 or negative. To assess the prognostic value of drug differential genes, a univariate COX risk regression analysis of endometrial carcinoma tumor group data was performed using R package ‘survival’, aiming to screen out drug differential genes significantly associated with survival. To avoid the overfitting problem, a least absolute shrinkage and selection operator (LASSO)-penalized cox regression analysis was performed using R package “glmnet”. Later, a prognostic model was established by multivariate COX risk regression analysis, and two prognosis-related DR-DEGs were finally acquired. Thereafter, the risk score was calculated for each patient using the formula: Risk score = ∑X λ*coef λ. Wherein, X λ stands for the relative expression level of the normalized differential genes for each drug; coef λ for coefficient. Patients in the tumor group were classified into high-risk and low-risk groups based on the median risk score. To determine the role of risk scores in the prognostic model of endometrial carcinoma patients, we performed a separate analysis of overall survival (OS) between high and low risk groups and genes with prognosis-related drug differences, and displayed it with Kaplan–Meier curves. In addition, the “Rtsne” package function of R software was used for principal component analysis (PCA) and t-SNE test. The groups were visualized to explore the distribution of different groups. R package “survivalROC” was used to perform time-dependent receiver operating characteristic (ROC) analysis, so as to test the specificity and sensitivity of the survival prognostic model.

### Functional enrichment analysis and immune infiltration analysis of differential genes in high and low risk groups

Differential expression analysis of genes in high and low risk groups was performed using the “limma” package to explore the possible causes of survival differences between high and low expression groups. The filter condition provided: |logFC|> 1; Threshold value for FDR (BH) after correction P.adj < 0.05. Differential genes between the high and low risk groups were obtained. Functional analysis of the differential genes was also performed using the “ClueGo” plugin in cytoscape (version 3.7.2). Through single-sample gene set enrichment analysis (ssGSEA) in R-software “GSVA” package, the infiltration scores of 16 immune cells and the activity of 13 immune-related collaterals were calculated.

#### Experimental preparation

Ishikawa cells used in the experiment were provided by Dr. Zhang Xiaodan. 96well plates, Beyotime cck8 kit, fetal bovine serum and dmem were purchased by gibco, and 6-well plates were purchased from corning company.

#### CCK8 assay

Cytotoxicity was determined by using CCK8 assay. After centrifugation, log-growing ishikawa endometrial carcinoma cells were inoculated in 96-well plates, containing about 1 × 104 cells per well, with PBS added to the peripheral wells to prevent marginal effects. After the cell adhered to the wall, the cell supernatant was discarded and Puerarin solutions with concentration gradient (0, 10, 25, 50, 75, 100, 150, 200, 250, 300uM) were added to continue the culture. At least 3 multiple wells were set at each concentration, and the experiments were repeated 3 times. After 24 h, 10 μL of CCK8 solution was added to each hole and cultured at 5%CO2 and 37 ℃ constant temperature incubator for 1 h. It was taken out to immediately measure the absorbance at 450 nm with an enzyme labeling instrument.

#### Cell wound scratch assay

Ishikawa endometrial cancer cells were seeded into 6-well plates at a density of 10^6 cells per well and cultured until the density reached 85%. The floating cells were gently cleaned and serum-free medium containing 0, 50 and 100 uM puerarin was added. Wound area was measured 24 h later to assess cell migration.

#### q-PCR experiment

Cells were inoculated in a six-well plate to 80% of confluence. The original medium was replaced with complete DMEM containing 0,25uM of Puerarin for intervention. According to the instructions of RNA extraction kit of FeJet Biotech, RNA was extracted in the six-well plates of blank group (cultured in the complete DMEM for 24 h) and drug group (complete DMEM containing 25uM Puerarin) in six-well plates after 24 h. The Genecopoeia reverse transcription kit was used for cDNA synthesis under 25℃, 5 min; 42℃, 15 min; 85℃, 5 min; and 4℃, holding setup program. After acquiring cDNA, GapDh was used as an endogenous control. The PCR reaction system is as follows: Premix Ex Taq (loading dye Mix) 10 μl,, 0.5 μl each in the upstream and downstream primers, cDNA 1 μL, ddH2O supplemented to the total system 20 μl. Reaction conditions: Pre-denaturation under 95℃ for 30 s; denaturation under 95℃ for 30 s; annealing under 60℃ for 30 s; extension under 65℃ for 1 min. The reaction was ended after 35 cycles under 65℃ with final extension of 5 min for observation. The primers for real-time PCR were as follows:

- PIM1: forward: GAGAAGGACCGGATTTCCGAC; reverse: CAGTCCAGGAGCCTAATGACG.
- BIRC5: forward: GGAGGGGACGTTGACCTTC; reverse: TTTTGTGGTAAGAGAGCTGACG.
- GAPDH was used as reference control. forward: GCACCGTCAAGGCTGAGAAC; reverse: TGGTGAAGACGCCAGTGGA.

## Results

### Drug-related differentially expressed genes (DR-DEGs) were obtained

**Fig. 1:**
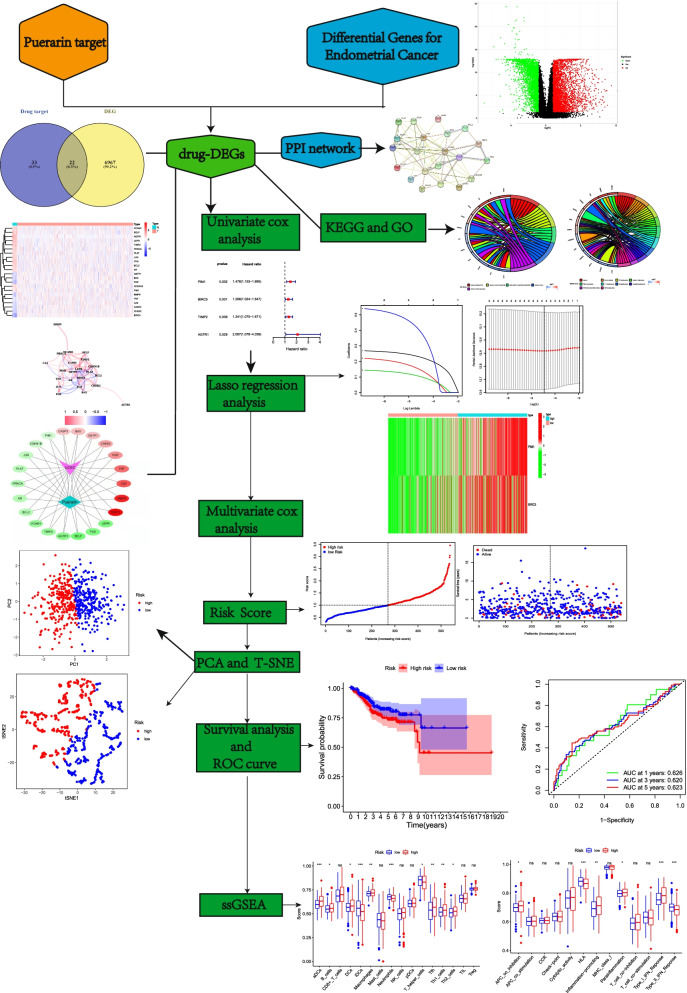
Flow chart. The workflow of data analysis in this study

**Fig. 2:**
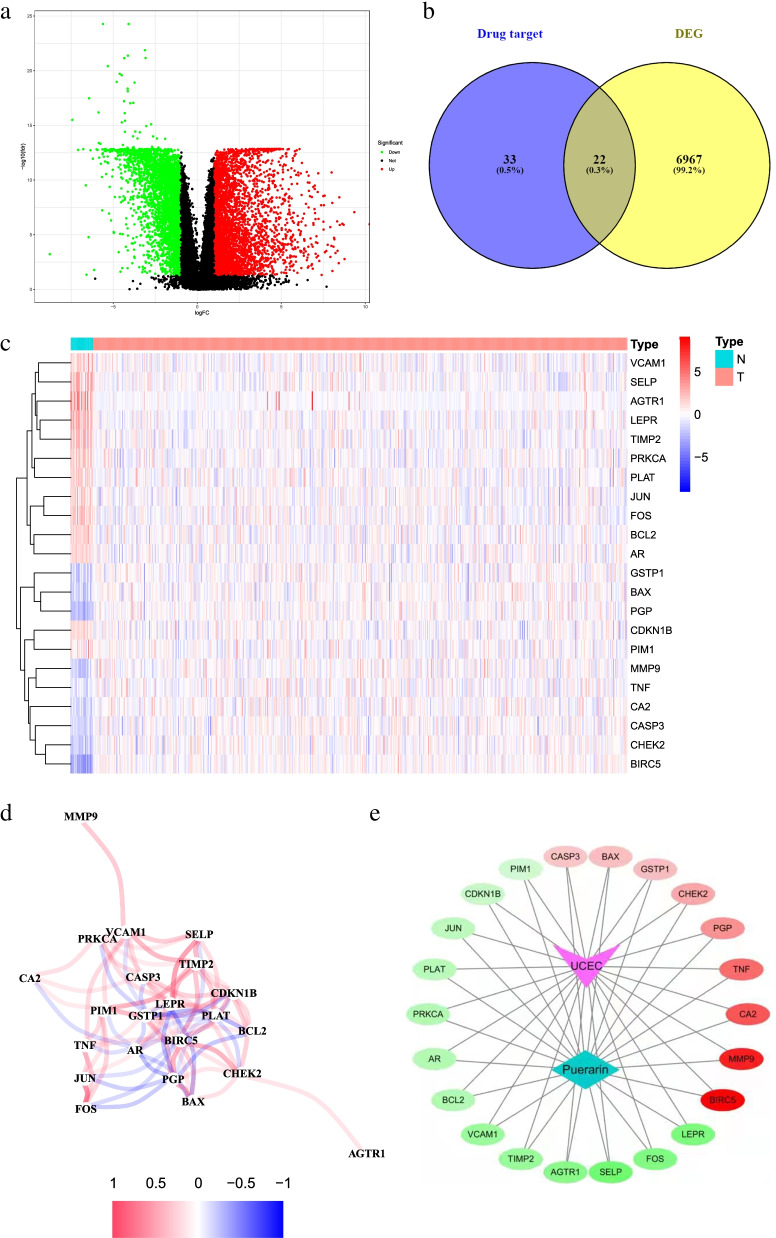
Drug-related differential genes of endometrial cancer and their interactions. **a** Volcano plot of 6989 differentially-drug genes in endometrial cancer; **b** Venny plot of 22 drug-related differential genes (DR-DEGs); **c** Heatmap of 22 DR-DEGs (N represents normal group, T represents tumor group; red represents high gene expression, blue represents low gene expression, and the depth of color represents the level of expression); **d** The relationship between 22 DR-DEGs (red line is Positive correlation, blue line is negative correlation, and the depth of color represents the strength of the correlation); **e** Disease- target-drug network diagram (red indicates gene up-regulation, green indicates gene down-regulation, and color depth represents the degree of regulation)

The analysis flow chart is shown in Fig. 1. We obtained a total of 55 drug target genes by searching the molecular targets of puerarin drugs in the TCMSP database and transforming and standardizing them with the uniport database. In addition, 6989 DEGs were obtained from 23 normal group samples and 522 tumor group samples genes obtained from TCGA website using R software “limma” package, and the results are shown in the volcano plot in Fig. 2a. The intersection was taken using venny2.0 to obtain 22 DR-DEGs (Fig. 2b). These RNA expression levels were shown in the heat map in Fig. 2c (highly expressed genes in red and lowly expressed in green), and an inter-gene expression diagram was established (Fig. 2d). Among them, 13 genes were upregulated in the normal group (VCAM1, SELP, AGTR1, LEPR, TIMP2, PRKCA, PLAT, JUN, FOS, BCL2, AR, CDKN1B, PIM1) and 9 genes were upregulated in the tumor group (GSTP1, BAX, PGP, MMP9, TNF, CA2, CASP3, CHEK2, BIRC5). The interaction between drug small molecules and DR-DEGs targets was visualized by cytoscape (version3.7.2) software, and the drug target—disease interaction network was constructed (Fig. 2e).

### Protein–protein interaction network and enrichment analysis

**Fig. 3:**
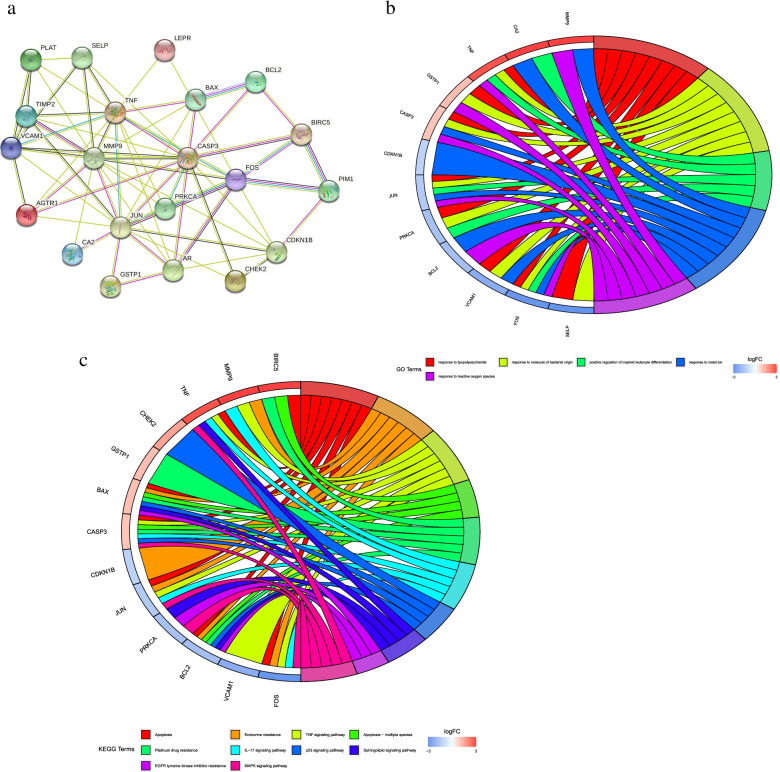
PPI Protein–protein interaction networks and GO, KEGG enrichment analysis. **a** PPI Protein–protein interaction networks; **b** GO enrichment analysis diagram of 22 DRG -DEGs; **c** KEGG enrichment of 22 DRG -DEGs Analysis chart

To observe the relationship between 22 DR-DEGs target proteins, we constructed the target protein–protein interaction network through STRING data. One gene with no interaction with the other target proteins was rejected, and the other 21 target proteins were all correlated (Fig. 3a). The four genes MMP9, TNF, CASP3 and JUN had the most connections in the protein interaction diagram, and had the closest relationship with other proteins. To explore the potential signaling pathways in 22 DR-DEGs, we performed a GO and KEGG enrichment analysis. GO enrichment analysis indicated that (Fig. 3b) DR-DEGs were enriched in “response to lipopolysaccharide, response to molecule of bacterial origin, positive regulation of myeloid leukocyte differentiation, response to metal ion, response to reactive oxygen species”. KEGG enrichment analysis expressed that DR-DEGs were enriched in “Apoptosis, Endocrine resistance and TNF signaling pathway” (Fig. 3c).

### The prognostic model was established and validated

**Fig. 4:**
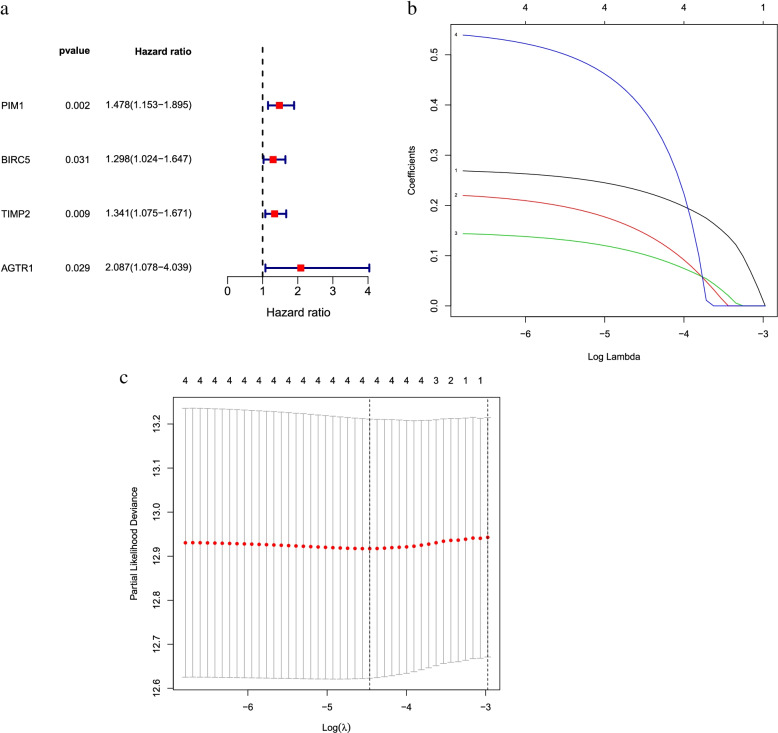
Selection of DR-DEGs associated with endometrial cancer prognosis by regression analysis. **a** Forest plot of 4 DR-DEGs associated with endometrial cancer survival was constructed by univariate regression analysis; **b** LASSO of 4 genes Analysis coefficient spectrum distribution; **c** Cross-validation of optimal parameter selection in LASSO regression

To establish a prognostic model associated with endometrial carcinoma, we performed a univariate COX analysis on the expression levels of 22 DR-DEGs and their clinical data. The analysis suggested four genes associated with survival in endometrial cancer (Fig. 4a). LASSO COX regression analysis of these four DR-DEGs showed that these genes performed stable and well (Fig. 4b and c). To find the optimal results, the four DR-DEGs were analyzed by multivariate COX regression that resulted in two genes associated with endometrial carcinoma—PIM1 and BIRC5.

**Fig. 5:**
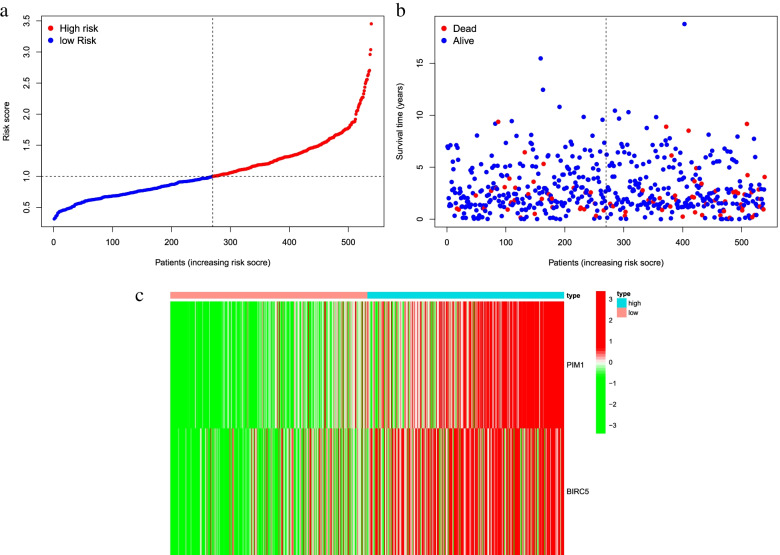
Characteristics of prognostic genes. **a** Distribution map of patients in high and low risk groups based on risk score; **b** Survival status map of patients in high and low risk groups (left side of the dotted line, low risk group; right side of the dotted line, high risk group); **c** Heatmap of DR-DEGs associated with prognostic features of endometrial cancer

**Fig. 6:**
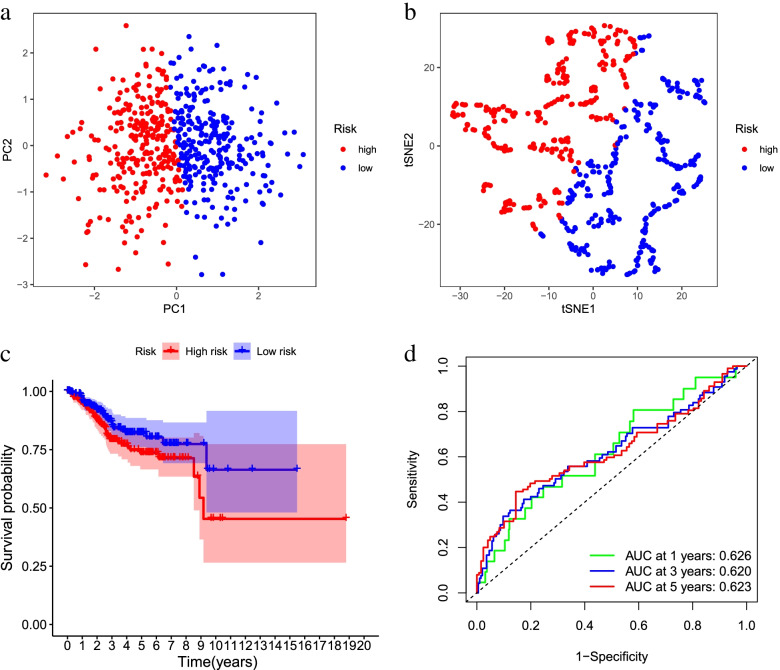
Prognostic Analysis of the Characteristic Model of DR-DEGs. **a** PCA plot based on risk score; **b** t-SNE map based on risk score; **c** Kaplan–Meier analysis curve of prognosis related to high and low risk groups; **d** Time-dependent ROC curve, the area under the curve (AUC) at 1 year, 3 years, and 5 years is 0.626, 0.620, 0.623

The risk score was calculated for each patient, based on mRNA expression levels and the risk coefficients of the two DR-DEGs. Patients above the median score were divided into high-risk groups based on the median risk score, or otherwise into low-risk groups to predict the prognosis of patients with endometrial carcinoma (Fig. 5a). With PCA-analysis and t-SNE test (Fig. 6a-b), the patients with different risks were well divided into two groups, and the distribution direction of the patients in the high and low risk group was significantly different. The mortality rate in the high-risk group was higher than that in the low-risk group, and the survival rate was lower than that in the low-risk group (Fig. 5b). The heat map of gene expression of two DR-DEGs in high-risk and low-risk groups was constructed (Fig. 5c). The results showed that the expression of PIM1 and BIRC5 in high-risk group was higher than that in low-risk group, proving that they were risk genes. Kaplan–Meier analysis showed that survival was higher in the low-risk groups at different times than in the high-risk groups (Fig. 6c). Time-dependent ROC curves were used to assess the prediction effect of the prognostic model (Fig. 6d). The results indicated one-year AUC of 0.626, two-year AUC of 0.620 and three-year AUC of 0.623, suggesting the stable and good prediction effect of the prognostic model.

### Functional analysis

**Fig. 7:**
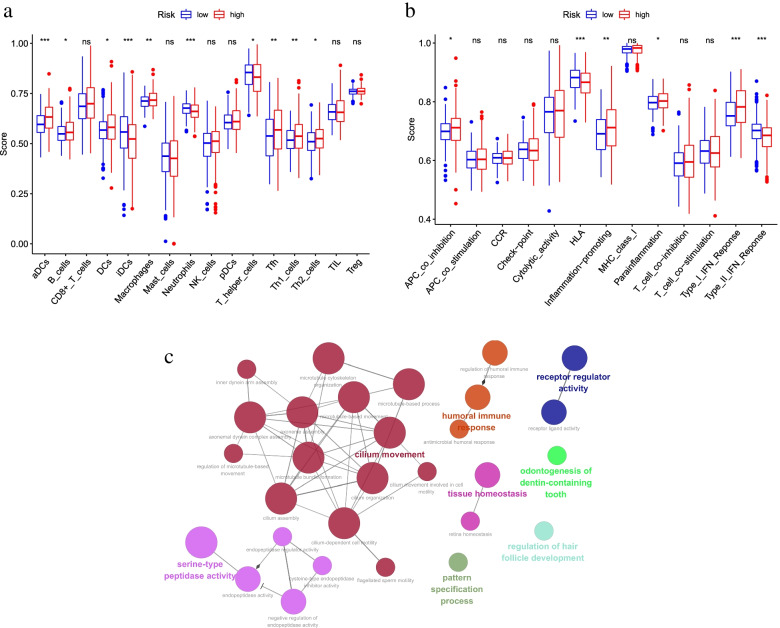
Comparison of immune infiltration analysis and functional analysis of differentially expressed genes between high and low risk groups. **a** Score map of 16 immune cell infiltration enrichment analysis in high and low risk groups; **b** Score map of 13 immune pathways related activity in high and low risk groups; **c** Analysis map of differential gene function between high and low risk groups

Functional analysis of differentially expressed genes in high-risk and low-risk groups was performed using ClueGO plug-in in Cytoscape software (3.7.2). The results of functional analysis referred to Fig. 7c.

### Immune cell and immune function analysis

Based on the classification of high and low risk groups, we conducted a single-sample gene enrichment analysis (ssGSEA), and obtained the enrichment scores for 16 immune cell infiltration and the related activity of 13 immune pathways (Fig. 7a-b). Significant different scores were found in the enrichment analysis of most immune cells (e. g., aDCs, iDCs, Neutrophils, etc.), where iDCs and Neutrophils were less infiltrated in the high-risk group and aDCs were less infiltrated in the low-risk group. At the same time, the differences in immune pathways (HLA, Type_I_IFN_Reponse, Type_II_IFN_Reponse) were more significant in the high and low risk groups. The high risk group obtained a higher score in Type_I_IFN_Reponse pathway and a lower score in Type_II_IFN_Reponse and HLA pathway.

#### CCK8 Results

**Fig. 8:**
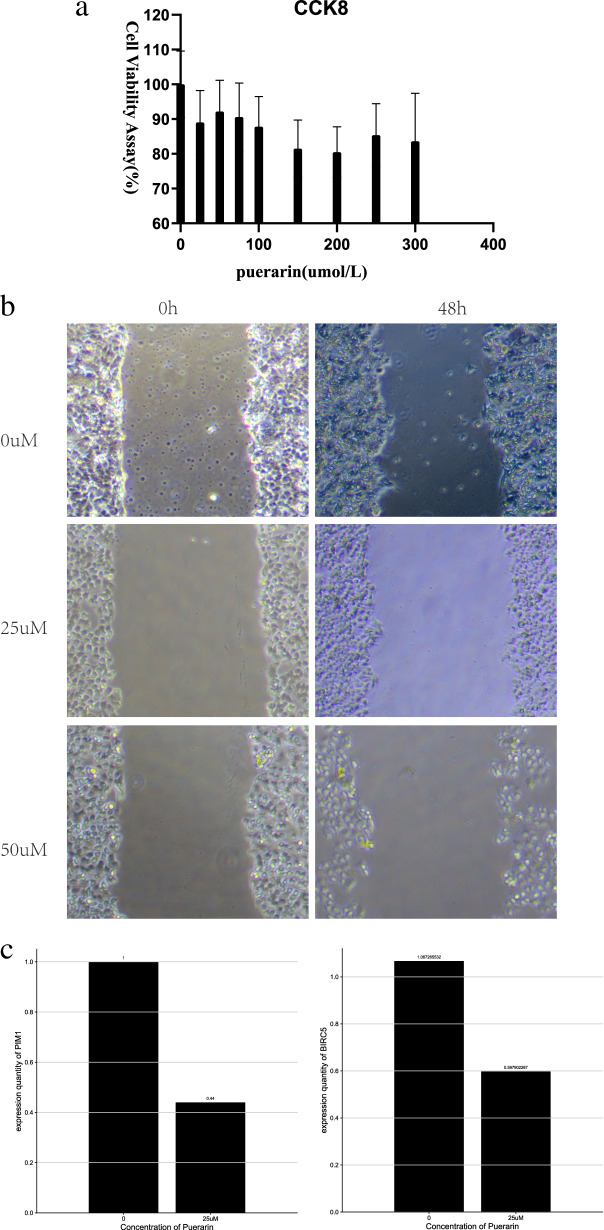
Experimental verification. **a** CCk8 cytotoxicity test; **b** Wound Healing Assay; **c** q-PCR

To clarify the toxic effects of puerarin on ishikawa, the effects on cells at different concentrations were examined. As shown, puerarin had an insignificant effect on the survival of ishikawa endometrial carcinoma cells. At experimental concentrations, puerarin significantly impaired ishikawa cytotoxicity in endometrial carcinoma cells(Fig. 8a).

#### Cell wound scratch assay results

In different concentrations of puerarin (0, 50, 100 uM), the wound healing rate showed a negative correlation with drug concentration (Fig. 8b).

#### q ‑PCR result

To further confirm these results, we compared the expression of PIM1 and BIRC5 genes in the control and drug groups by q-PCR, and the mRNA levels of both PIM1 and BIRC5 were significantly reduced in 25uM puerarin compared to the control endometrial cancer cells. (Fig. 8c).

## Discussion

Endometrial carcinoma is a common tumor in the female reproductive system with rising incidence and mortality rate in recent years, making it become an important factor affecting women’s health [22]. Apart from much progress made in the biological mechanistic studies of endometrial carcinoma, there are still many controversies in the treatment of endometrial carcinoma, including the evaluation of lymph node excision and the choice of adjuvant treatment or chemotherapy [23]. Therefore, it is particularly important to explore new drug small molecules for treating endometrial carcinoma and to establish new differential gene prognostic models. As a small Chinese medicine molecule extracted from pueraria lobata, puerarin has been widely used in clinical treatment with its anti-cancer effect [24]. Whether it can be applied in the treatment of endometrial carcinoma has not been well studied. In view of this, we explored the pathway and potential mechanisms of treating endometrial carcinoma with bioinformatics technology, verified it with experiments, and constructed a prognostic model.

To explore the potential mechanisms of puerarin in the treatment of endometrial carcinoma, we performed a GO and KEGG enrichment analysis on its disease drug targets. GO enrichment analysis suggested that it was significantly enriched in the “response to LPS” and “response to bacterial molecular sources”. Cytowall peptides, as a known component of peptidoglycan, can participate in hematopoietic processes and stimulate the production of colony-stimulating factors for the treatment of tumor cells [25]. At the same time, LPS functions as a major component of the cell wall of Gram-negative bacteria, which can be recognized by TLR-4 and alter the immune response in different cancer patients [26, 27]. Interestingly, TLR-4 is also present in endometrial dendritic cells, monocytes, and macrophages [28]. Therefore, it is assumed that puerarin may regulate the immune response of immune cells in the endometrium through “LPS” and “bacterial molecule” pathways for the treatment of endometrial carcinoma.

KEGG enrichment analysis reveals that Interleukin-17(IL-17) signaling pathway is one of its important pathways. Many studies have shown that IL-17, as an important cytokine for promoting cell growth, can promote tumor formation by maintaining the inflammatory microenvironment [29]. Wang et al. discovered that by studying IL-17 knockout mice, IL-17 disruption reduced tumorigenesis, which may be associated to STAT3 activation in the tumor microenvironment [30]. Alternatively, IL-17 can also upregulate the expression of multiple provascular growth factors to lead to the generation of new blood vessels and maintain and promote tumor growth [30, 31]. We therefore hypothesized that IL-17 may be closely related to endometrial carcinoma formation by regulating chemokines and cytokines in gynecological tumors. The regulation of IL-17 signaling pathway may be a potential mechanism for puerarin in the treatment of endometrial carcinoma [32]. Studies have shown that the glucose uptake and glycolysis of glucose were increased through MAPK pathway, thereby promoting the proliferation of endometrial carcinoma cells [33]. Bai et al. concluded that ERK1/2, as one of MAPK pathway kinases, is highly expressed in cervical cancer tissues. The development of cervical cancer cells can be promoted by the modulation of c-FOS and cJUN proteins. After using ERK1/2 inhibitor, the growth of cervical cancer cells was significantly inhibited and the apoptosis of cells was accelerated [34]. In addition, estrogen can also promote the development of endometrial carcinoma cells by activating MAPK pathway [35–37]. Interestingly, it is found that puerarin could inhibit MAPK through reviewing the literature. For example, puerarin can improve cyanin-induced chronic pancreatitis by inhibiting MAPK signaling, as concluded by Zeng et al. [38] As discovered by XIAO, et al., through inhibiting TRAF6/ ros-dependent MAPK/NF-κB signaling pathway, puerarin can inhibit the formation of osteoclasts and reduce osteocast-associated bone mass loss in mice with oophorectomy (OVX) -induced osteoporosis [39]. Alternatively, puerarin can also inhibit the fine particulate matter-induced proliferation of vascular smooth muscle cells by inhibiting MAPK signaling pathway [40]. We therefore hypothesized that MAPK signaling promotes the appreciation of endometrial carcinoma cells by increasing glucose uptake, regulation of c-FOS and cJUN proteins, and being activated by estrogen. Puerarin restricts the development of endometrial carcinoma in a way that suppresses MAPK pathway, which is a potential mechanism to resist against the proliferation of endometrial carcinoma cells.

Using multivariate COX regression analysis, two genes associated with endometrial carcinoma prognosis—PIM1 and BIRC5 were obtained, both of which were high-risk genes. Pro-viral integration site for Moloney murine leukemia virus1 (PIM1) is a serine-coded / arginine-coded proto-oncogene [41, 42]. PIM1 gene is considered to be the causative genes for epithelial origin cancer line diseases such as prostate and breast cancer as well as hematological tumors such as diffuse large B-cell lymphoma [43–45], which is closely related to the risk of cancer occurrence. It is reported that PIM1 expression is closely related to lymph node metastasis and poor prognosis in lung adenocarcinoma and squamous cell carcinoma patients [46]. We regard it as a high-risk gene associated with the abnormal overexpression and prognosis in endometrial carcinoma cells. baculoviral IAP repeat contains5 (BIRC5), as the apoptosis inhibitor, may prevent the cell apoptosis through encoding “negative regulatory proteins”. The expression of BIRC5 increased sequentially from proliferative endometrium to endometrial hyperplasia to endometrioid adenocarcinoma [47]. It was found that BIRC5 acts to promote endometrial carcinoma progression and is overexpressed in both endometrial carcinoma and endometrial carcinoma cell lines [48, 49]. Overexpression of BIRC5 in endometrial carcinoma is an independent prognostic factor, as found by Zhao et al [50]. This was also demonstrated by the study of Chuwa et al. It was also found that BIRC5 knockout in endometrial carcinoma cells could promote cell apoptosis. This demonstrates that BIRC5, besides serving as an independent prognostic factor, may also serve as a new treatment target for endometrial carcinoma [51].

ssGSEA was used to analyze the enrichment scores of the 16 immune cell infiltrates obtained and the associated activity of the 13 immune pathways. Dendritic cells (DCs), as specialized antigen-presenting cells, are responsible for the activation of specific T-cells and immune tolerance [52]. iDcs are immature dendritic cells that are mainly found in peripheral tissues. We found that iDCs scored lower in the high-risk group. Hasumi K et al. found that patients with advanced cancer were alleviated by injecting iDCs [53]. Therefore, we speculate that iDCs are positively associated with patient survival, and their low expression in high-risk groups may affect antigen presentation and T-cell activation in the tissues of endometrial carcinoma patients, thus affecting patient survival. Human leukocyte antigen (HLA) is associated with the presentation of the neoantigen and critical steps in the cytolytic T-cell response. Downregulation of HLA genes may also lead to reduced antigen presentation and immune evasion [54]. Downregulation of HLA is prevalent in a range of cancers and is closely related to its poor prognosis [55–57]. We therefore speculate that HLA is positively associated with tumor prognosis risk prevention. The activity analysis results of our immune pathway are consistent with our speculation that HLA scored low in the high-risk group and the difference was statistically significant.

It was experimentally verified that puerarin, in the experimental dose, did not show a significant toxic effect on the cells, for which we selected the experimental drug concentration under IC30 (0, 50, 100UM). The results of the wound healing experiments concluded that the cell migration ability of puerarin to endometrial carcinoma cell line Ishikawa appeared with increasing concentration. It is therefore reasonable to conclude that puerarin inhibition of cell migration capacity in endometrial carcinoma is not accomplished by prompting its cell death. This coincides to the response to lipopolysaccharide [58] in go analysis, EGFR tyrosine kinase inhibitor resistance [59–61] and MAPK signaling pathway in kegg analysis [62], which has been reported in the articles on the migratory ability of tumor cells.

## Data Availability

The data for this study come from The Cancer Genome Atlas (TCGA) database (https://portal.gdc.cancer.gov/). All the data in this paper support the results of this study.
